# Early experience with a rapid navigation system-assisted unilateral biportal endoscopic interbody fusion for lumbar spondylolisthesis

**DOI:** 10.3389/fsurg.2026.1781960

**Published:** 2026-03-11

**Authors:** Cheng Peng, Zhinan Ren, Jiansen Wang, Jing Zhang, Yukang Qin, Lei Yu, Guangduo Zhu, Yingjie Hao

**Affiliations:** Department of Orthopedics, The First Affiliated Hospital of Zhengzhou University, Zhengzhou, Henan Province, China

**Keywords:** intraoperative navigation, lumbar interbody fusion, lumbar spondylolisthesis, neurodecompression, unilateral biportal endoscopic technique

## Abstract

**Objective:**

This study aims to investigate the efficacy of a simple and effective two-dimensional integrated navigation-guided spinal endoscopic interbody fusion surgery for the treatment of lumbar spondylolisthesis.

**Methods:**

A retrospective analysis was conducted on the clinical data of patients with lumbar spondylolisthesis treated with or without navigation. Postoperative clinical data were collected, and pain levels were assessed using the Visual Analog Scale (VAS), while functional improvement was evaluated using the Oswestry Disability Index (ODI). At the final follow-up, x-ray imaging was used to measure disc height (DH), slip percentage (SP), lumbar lordosis (LL), and slip angle (SA) at the surgical segment. Interbody fusion status was assessed based on the Bridwell grading criteria.

**Results:**

ULIF surgery for lumbar spondylolisthesis with navigation assistance yields favorable early outcomes, including reduced surgery duration, fewer fluoroscopy instances, lower intraoperative blood loss, and less postoperative drainage volume (*P* < 0.05). Patients in the navigation group also show greater early postoperative improvements in low back pain (*P* < 0.05). No significant differences were observed between the two groups in other evaluation indicators (*P* > 0.05).

**Conclusion:**

Compared with the non-navigation group, the ULIF procedure guided by the two-dimensional integrated navigation system—which is simple and effective—can provide better early relief of low back pain in patients with lumbar spondylolisthesis, while also shortening operative time, reducing the number of fluoroscopy instances, minimizing intraoperative blood loss, and decreasing postoperative drainage volume. This surgical approach demonstrates great potential for widespread clinical application.

## 1 Introduction

Lumbar spondylolisthesis (LS) refers to the displacement between adjacent vertebrae, typically resulting from factors such as intervertebral disc degeneration, spondylolysis, and insufficient stability of ligaments and surrounding muscles. This condition often manifests as clinical symptoms, including low back pain and radiating pain or numbness in the lower limbs (1–4). Currently, interbody fusion surgery is among the most commonly employed surgical treatments for LS (5–7). The conventional approach involves open laminotomy and fusion, which requires extensive removal of the lamina and causes significant damage to muscle tissue. This approach is associated with increased risks of postoperative chronic low back pain, muscle atrophy, and a higher incidence of complications (8). Minimally invasive transforaminal lumbar interbody fusion (MIS-TLIF) is widely adopted for mild LS due to its reduced soft tissue damage and quicker recovery times. However, this technique still necessitates exposure of the paravertebral muscles and lamina, leading to substantial structural damage (9, 10). In 1996, De Antoni et al. (11) introduced a dual-approach endoscopic technique, which was later termed the unilateral biportal endoscopic (UBE) technique by Heo et al. (12) in 2017. This innovation enabled successful endoscopic lumbar fusion, yielding promising clinical outcomes.

However, the application of the UBE technique in interbody fusion surgery also presents several challenges. Although the UBE technique provides a dual-channel endoscopic view, surgeons may still face limitations in visibility, particularly when navigating complex anatomical structures. This restricted view can result in inadequate identification of critical nerves or blood vessels, thereby increasing the risk of intraoperative injuries. Consequently, it is crucial for surgeons to attain proficiency in operating the dual-channel endoscope. For novice surgeons, the steep learning curve associated with this technique may adversely affect the safety and efficacy of the procedure. Furthermore, the intricate dual-channel operations and precise techniques required in UBE surgery often leads to prolonged surgical durations, especially during the initial implementation of this technology. This extended operating time may heighten the risks associated with prolonged anesthesia exposure for patients (13, 14).

To address the aforementioned challenges, we employed an intraoperative two-dimensional navigation system in place of traditional C-arm fluoroscopy for spinal endoscopic interbody fusion surgery. This navigation system, based on the C-arm, reconstructs the surgical site by capturing images and transmitting them to the navigation interface for automatic matching. By integrating advanced positioning technology, the system aids surgeons in determining optimal entry points and trajectories for pedicle screws, as well as selecting the most appropriate screw sizes, thereby improving the precision and safety of the procedure (15, 16). A key advantage of this C-arm-based two-dimensional navigation system is its ease of operation, which minimizes the learning curve for surgeons. Additionally, the system is quick, convenient, and compatible with most existing hospital equipment, eliminating the need for high-end resources and facilitating broader adoption. Meanwhile, compared with isolated lumbar disc herniation or simple lumbar spinal stenosis, lumbar spondylolisthesis is frequently associated with vertebral slippage, altered pedicle orientation, segmental instability, and distortion of anatomical landmarks. These factors markedly increase the technical difficulty of pedicle screw placement and cage positioning, particularly in minimally invasive and endoscopic fusion procedures. Consequently, lumbar spondylolisthesis provides a more challenging clinical scenario for evaluating the potential advantages of intraoperative navigation. We conducted a retrospective analysis of patients who underwent navigation-guided unilateral biportal endoscopic fusion (ULIF) surgery in the spinal surgery department of our center to evaluate its clinical efficacy, aiming to provide data support for research on navigation-guided treatment of lumbar spondylolisthesis.

## 2 Methods

### 2.1 Study design and oversight

We collected data from patients with LS who underwent ULIF surgery in the department of spine surgery at our hospital between June 2022 and October 2023, and screened them according to the following criteria.

Inclusion criteria: (1) patients with LS who underwent ULIF guided by two-dimensional navigation or non-navigation techniques; (2) imaging findings indicating single-level lumbar degenerative spondylolisthesis, Meyerding grade (17) I or II, with lumbar instability and secondary spinal stenosis; (3) symptoms including low back pain and/or intermittent claudication and/or radiating pain in the lower limbs, with no significant improvement after at least 3 months of conservative treatment, and poor quality of life, meeting surgical indications; (4) availability of complete symptom scoring and imaging follow-up data, with the last follow-up at least 12 months postoperatively. Cases not meeting these criteria were excluded from the study.

Exclusion criteria: (1) severe lumbar scoliosis (Cobb angle > 30°); (2) history of lumbar or retroperitoneal surgery; (3) presence of lumbar fractures, spinal infections, or tumors; (4) severe hip or knee joint diseases, or history of hip or knee joint replacement; (5) presence of osseous spinal canal stenosis; (6) severe osteoporosis (T-score <−2.5).

**Figure 1:**
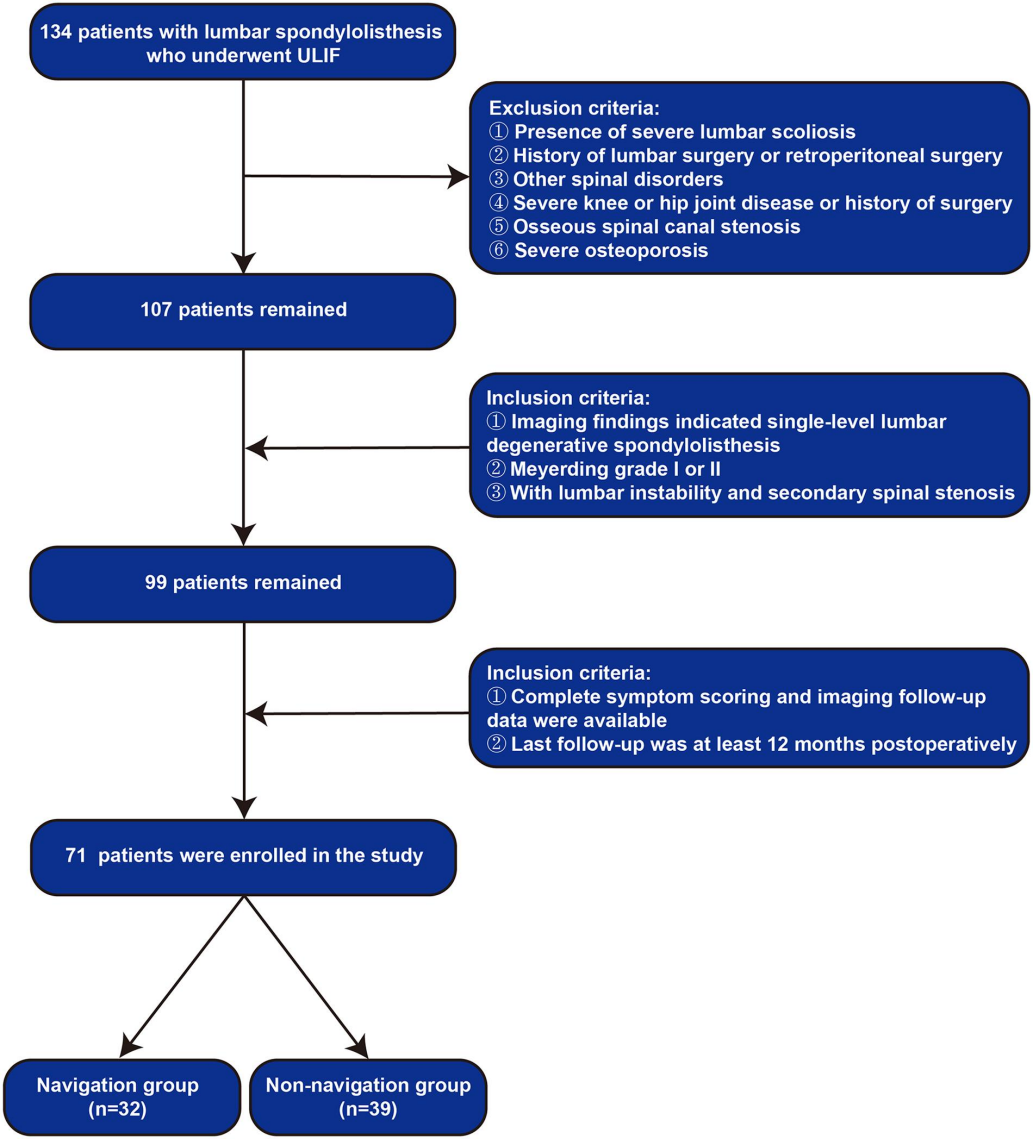
Patients screening process.

71 patients were selected for the study, with 32 in the navigation group and 39 in the non-navigation group (Figure 1). Data collected included age, gender, height, weight, Meyerding grading, and the lumbar segments treated. This study was approved by the Ethics Committee of the First Affiliated Hospital of Zhengzhou University (Approval No. 2022-KY-1783-001).

### 2.2 Navigation equipment and surgical procedure

**Figure 2:**
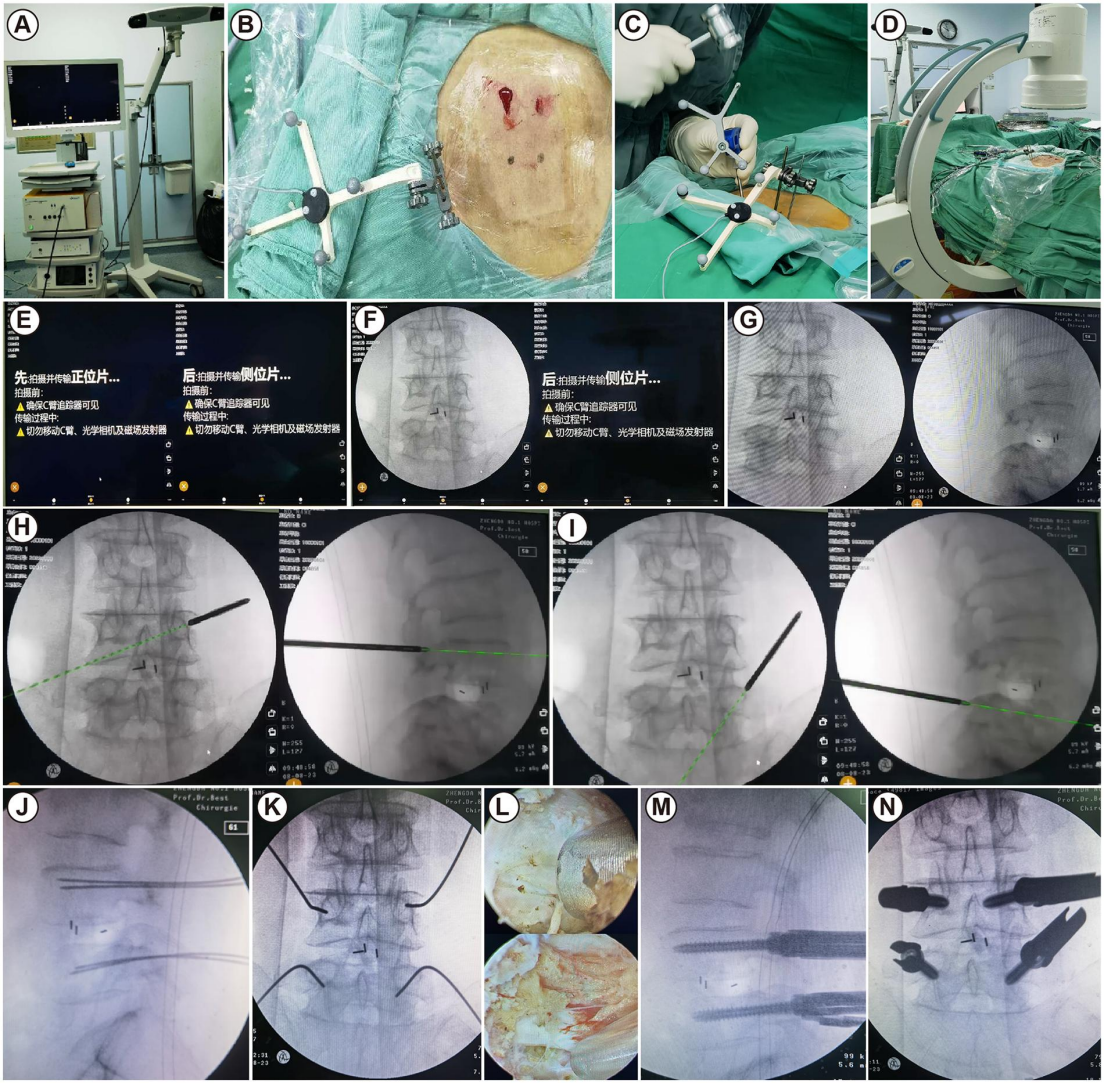
Surgical steps for unilateral bilateral endoscopic interbody fusion guided by navigation. **(A)** Two-dimensional navigation equipment; **(B,C)** installation of image-guided dynamic navigation reference frame; **(D)** automatic image acquisition by C-arm; **(E–G)** automatic upload of anteroposterior and lateral lumbar x-ray images for analysis; **(H,I)** pedicle puncture of L4 and L5 guided by navigation; **(J,K)** navigation-guided placement of a lumbar localization guidewire; **(L)** nerve decompression using UBE technique; **(B)** implantation of pedicle screws in the L4 and L5 vertebrae.

We performed the surgeries using this two-dimensional C-arm–based navigation system, which integrates both optical and electromagnetic tracking technologies. Through a unified registration and coordinate mapping framework, the system enables seamless switching between optical and electromagnetic navigation modes in the intraoperative environment, thereby combining the advantages of optical navigation in terms of stability and accuracy with the unique adaptability of electromagnetic navigation for tracking flexible instruments under complex, non-line-of-sight conditions. The software algorithms and interactive workflow of the system were also developed with direct involvement of clinical surgeons, with particular emphasis on optimizing key components such as image registration, instrument tracking, and real-time feedback to ensure operability and robustness in real-world surgical settings. Following system development, the research team systematically validated the registration error and practical application accuracy of the navigation system, clearly distinguishing between theoretical engineering-level errors and composite errors encountered in clinical operative environments. This validation process provided reliable evidence of safety and accuracy to support subsequent clinical application (18). The navigation equipment is shown in Figure 2A.

The surgeries for both patient groups were performed by the same team from the Department of Spine Surgery at our hospital. After the patient was under general anesthesia, they were placed in the prone position, with cushions placed on both sides of the chest and abdomen to keep the abdomen suspended. The C-arm fluoroscopy was used to locate the surgical interspace and bilateral pedicles, and the locations were marked on the skin. After routine disinfection, the surgical drapes were arranged in a “U” shape centered around the incision to ensure that irrigation fluid could drain smoothly from the surgical area. Next, the image-guided dynamic navigation reference frame was securely fixed to the iliac bone to ensure no movement during surgery (Figures 2B,C). The position was adjusted to ensure that the lumbar vertebrae of the surgical segment were centered in both the anteroposterior and lateral images. During fluoroscopic scanning, the C-arm automatically rotated 180°, and the captured images were immediately transmitted to the navigation system for reconstruction to obtain multiplanar images (Figure 2D). After registering the navigation probe, drill, and tracking pins, they could be recognized and tracked in real-time by the system during surgery (Figures 2E–G). With the assistance of navigation guidance, the entry points and angles for the pedicle screws of the vertebral body in the surgical segment were determined based on real-time two-dimensional images (Figures 2H,I). The optimal entry points and trajectories for each screw were identified using the anatomical structures of the surgical area provided by the images generated by the navigation system. Subsequently, guided by the navigation system, a guidewire was inserted into the pedicle of the vertebral body at the surgical segment to complete the screw channel preparation. During this process, real-time monitoring was conducted using the registered probe and other relevant tools (Figures 2J–K). Next, decompression surgery was performed (Figure 2L). A horizontal incision was made at the projection of the upper and lower pedicles on the side with more severe neurological symptoms. The surgeon's left side was used as the observation channel, and the right side as the operation channel. The incision lengths were 1 cm and 2 cm, respectively. The surgical site was incised layer by layer, and the muscles overlying the spinous process and lamina were bluntly dissected using a periosteal elevator. After connecting the endoscopic system (IMAGE1S camera system, KARL STORZ, Germany), further exposure was achieved using a radiofrequency probe. A bone knife was used to remove part of the lamina, inferior articular process, and the medial portion of the superior articular process. The nucleus pulposus was completely removed using a nucleus forceps, and an intervertebral distractor was used to remove intervertebral tissue. The cartilage endplates were scraped, and autologous bone granules and allograft bone strips were implanted into the intervertebral space, followed by the insertion of an expandable fusion cage (Shanghai RuiZhi Medical Devices, dual-plane expandable fusion cage, registration number: 20173464726). Pedicle screws were then inserted, and the connecting rods and locking caps were installed. The upper vertebral body was repositioned by pulling, and the proximal locking caps were tightened to secure the fixation. A re-exploration confirmed that the dura mater and bilateral nerve roots were not significantly compressed. Fluoroscopy showed satisfactory positions of the screws, rods, and fusion cage (Figures 2M,N). Finally, the incision was disinfected and sutured.

The main difference in surgical steps between the non-navigation and navigation groups is the placement of the guidewire. After the patient is prepped and draped, the guidewire is inserted into the pedicle of the vertebral body in the surgical segment based on the surgeon's experience and C-arm fluoroscopy. Following decompression under spinal endoscopy and the insertion of the fusion cage, pedicle screws are placed using the guidewire and intraoperative C-arm fluoroscopy. All other steps are identical to those in the navigation group.

### 2.3 Postoperative interventions and measurement of evaluation indicators

Both groups of patients received standard postoperative treatment, including analgesia, dehydration, and neurotrophic therapy. Patients were then assisted in wearing braces and encouraged to begin functional exercises as soon as possible. Follow-up lumbar spine x-rays and CT scans were performed on the third postoperative day, and at 1, 3, 6, and 12 months postoperatively. Surgical duration, number of intraoperative C-arm fluoroscopy views, postoperative drainage volume, length of hospital stay, intraoperative blood loss, and the occurrence of major complications were recorded and compared. Intraoperative blood loss was estimated by subtracting the total volume of physiological saline infused into the incision from the total volume of fluid drained during surgery (19–21).

**Figure 3:**
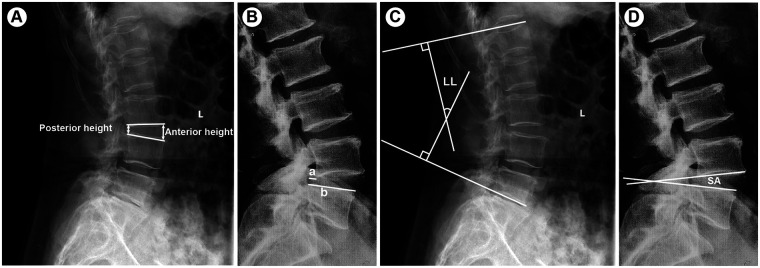
Radiological measurements of lumbar parameters on standing lateral x-ray images. **(A)** Disc height (DH) was calculated as the average of the anterior and posterior intervertebral disc heights at the surgical segment; **(B)** slip percentage (SP) was calculated as the ratio of the anterior displacement of the slipped vertebra to the anteroposterior length of the inferior vertebral body; **(C)** lumbar lordosis (LL) was measured using the Cobb method between the superior endplate of L1 and the superior endplate of S1; **(D)** slip angle (SA) was defined as the angle between the inferior endplate of the slipped vertebra and the superior endplate of the subjacent vertebra.

The Visual Analogue Scale (VAS) was used to assess the severity of low back pain and leg pain in both patient groups before surgery, and at 3 days, 1-, 3-, 6 months, and 1 year postoperatively (where 0 indicates no pain and 10 indicates severe pain). Additionally, the Oswestry Disability Index (ODI) was evaluated, with a maximum score of 50, where a higher score indicates more severe functional impairment (22). The percentage score was calculated based on the patient's score relative to the total possible score. At the last follow-up, interbody fusion status was evaluated based on lumbar x-rays and CT scans, and graded according to the Bridwell interbody fusion classification, with grades I and II indicating fusion and grades III and IV indicating non-fusion (23). Lumbar lordosis (LL), slip angle (SA), slip percentage (SP), and disc height (DH) at the surgical segment were also measured (24, 25). All radiological parameters were measured on standing lateral lumbar x-ray images obtained preoperatively and at follow-up, using the measurement methods illustrated in Figure 3. All measurements were independently performed by two experienced spine surgeons who were blinded to the patient group allocation.

### 2.4 Statistical analysis

Statistical analysis was performed using SPSS 27.0 (IBM, USA). Measurement data were normally distributed and presented as mean ± SD. Independent sample *t*-tests were used to compare clinical evaluation indicators between the two groups. Paired *t*-tests were used to compare imaging parameters before and after surgery. Repeated measures ANOVA was employed for comparing VAS and ODI scores between the two groups; if the sphericity assumption was not met, the Greenhouse–Geisser method was used for correction. Bonferroni correction was applied for comparisons at different time points within the same group, and multifactorial ANOVA was used for comparisons between different groups at the same time point. For count data, chi-square tests were used for comparing non-ranked data between the two groups, while Mann–Whitney *U* tests were used for ranked data. *P* < 0.05 was considered statistically significant.

## 3 Results

### 3.1 Baseline characteristics and clinical indicators of the patients

The comparison of data between the navigation and non-navigation groups is shown in Table 1. In terms of baseline characteristics, there were no statistically significant differences in age, BMI, gender, Meyerding grade, and surgical segment between the two groups (*P* > 0.05). Regarding clinical data, the navigation group had significantly shorter operation time (114.7 ± 10.3 vs. 134.9 ± 16.5), fewer intraoperative C-arm fluoroscopy instances (3.9 ± 0.9 vs. 7.7 ± 1.1), less intraoperative blood loss (101.2 ± 27.9 vs. 138.9 ± 50.7), and lower postoperative drainage volume (131.9 ± 24.4 vs. 174.8 ± 43.9) compared to the non-navigation group (*P* < 0.05). However, there were no statistically significant differences in postoperative hospital stay (7.5 ± 1.7 vs. 7.7 ± 1.9) and the incidence of major complications one year postoperatively (6.3% vs. 7.7%) between the two groups (*P* > 0.05).

**Table 1:** Baseline characteristics and outcome data of patients.

Variable	Navigation (*n* = 32)	Non-navigation (*n* = 39)	*t* (*χ*^2^, *Z*) Value*	*P* value
Age (years)	63.5 ± 3.7	62.9 ± 6.9	0.465	0.643
Body mass index (kg/m^2^)	24.8 ± 1.5	24.5 ± 1.6	0.445	0.658
Gender			0.063	0.815
Male	13	17		
Female	19	22		
Meyerding grade			−0.893	0.372
I	18	26		
II	14	13		
Surgical segments			1.905	0.386
L3/4	1	2		
L4/5	17	26		
L5/S1	14	11		
Duration of operation (min)	114.7 ± 10.3	134.9 ± 16.5	−6.290	<0.001
Fluoroscopy during operation (times)	3.9 ± 0.9	7.7 ± 1.1	−5.659	<0.001
Estimated blood loss (mL)	101.2 ± 27.9	138.9 ± 50.7	−3.967	<0.001
Postoperative drainage (mL)	131.9 ± 24.4	174.8 ± 43.9	−5.214	<0.001
Length of hospital stay after surgery (days)	7.5 ± 1.7	7.7 ± 1.9	−0.486	0.628
Major complication	2/32 (6.3%)	3/39 (7.7%)	0.056	0.813
Bridwell interbody fusion rate	28/32 (87.5%)	33/39 (84.6%)	1.243	0.537

* The statistical values for Gender, Surgical segments, Major complications, and Bridwell Interbody Fusion Rate are represented as *χ*^2^ values; the statistical value for Meyerding grade is represented as a *Z* value, while the others are represented as t values.

In terms of major complications one year postoperatively, the navigation group had one patient with a superficial wound infection, which improved following antibacterial treatment and debridement after bacterial culture of the discharge. Another patient in this group was suspected of having a hematoma at the surgical site and underwent surgical exploration for hematoma removal. In the non-navigation group, one patient experienced cerebrospinal fluid leakage during the perioperative period, which resolved with conservative management, including bed rest and fluid replacement therapy. Additionally, one patient developed a superficial wound infection that improved with treatment, and another patient experienced a fracture of the internal fixation screws and rods one year postoperatively, requiring revision surgery.

**Figure 4:**
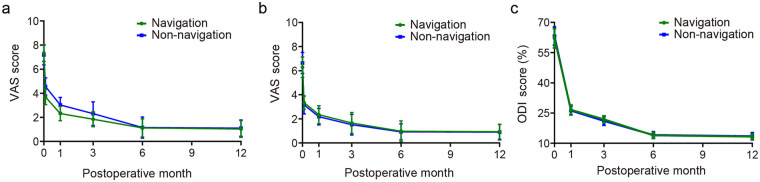
VAS and ODI scores for two groups of patients. **(a)** VAS scores for low back pain before and after surgery; **(b)** VAS scores for leg pain; **(c)** ODI scores expressed as a percentage before and after surgery.

The postoperative VAS scores for low back pain in both groups of patients (Table 2 and Figure 4A) significantly decreased over the follow-up period (*P* < 0.05). Notably, patients in the navigation group exhibited a more pronounced reduction in early low back pain scores (*P* < 0.05). The postoperative VAS scores for leg pain (Table 3 and Figure 4B) and ODI scores (Table 4 and Figure 4C) also showed significant decreases over the follow-up period (*P* < 0.05), but there were no statistically significant differences between the two groups (*P* > 0.05).

**Table 2:** VAS scores for low back pain in two groups of patients.

Group	Preoperative	Postoperative at 3 days	Postoperative at 1 month	Postoperative at 3 months	Postoperative at 6 months	Postoperative at 12 months
Navigation (*n* = 32)	7.3 ± 0.7	3.7 ± 0.6	2.3 ± 0.6	1.8 ± 0.6	1.1 ± 0.8	1.0 ± 0.7
Non-navigation (*n* = 39)	7.1 ± 0.8	4.6 ± 0.7	3.1 ± 0.6	2.3 ± 1.0	1.2 ± 0.9	1.1 ± 0.6
Statistic	Time *F* = 776.379, *P* < 0.001 Time*Group *F* = 6.092, *P* < 0.001 Group *F* = 14.642, *P* < 0.001					

**Table 3:** VAS scores for leg pain in two groups of patients.

Group	Preoperative	Postoperative at 3 days	Postoperative at 1 month	Postoperative at 3 months	Postoperative at 6 months	Postoperative at 12 months
Navigation (*n* = 32)	6.3 ± 0.8	3.3 ± 0.5	2.3 ± 0.7	1.7 ± 0.8	1.0 ± 0.8	0.9 ± 0.6
Non-navigation (*n* = 39)	6.6 ± 0.9	3.1 ± 0.7	2.2 ± 0.6	1.5 ± 0.7	0.9 ± 0.6	0.8 ± 0.5
Statistic	Time *F* = 604.611, *P* < 0.001 Time*Group *F* = 1.430, *P* = 0.213 Group *F* = 0.174, *P* = 0.678					

**Table 4:** ODI scores for two groups of patients*.

Group	Preoperative	Postoperative at 1 month	Postoperative at 3 months	Postoperative at 6 months	Postoperative at 12 months
Navigation (*n* = 32)	62.1 ± 4.8	26.6 ± 2.4	21.9 ± 1.7	13.9 ± 1.5	13.3 ± 1.2
Non-navigation (*n* = 39)	63.2 ± 4.5	26.2 ± 2.1	21.1 ± 2.1	14.1 ± 1.6	13.5 ± 1.7
Statistic	Time *F* = 4,333.670, *P* < 0.001 Time*Group *F* = 1.278, *P* = 0.281 Group *F* = 1.109, *P* = 0.742				

* ODI scores expressed as a percentage.

### 3.2 Imaging evaluation parameters of the patients

**Figure 5:**
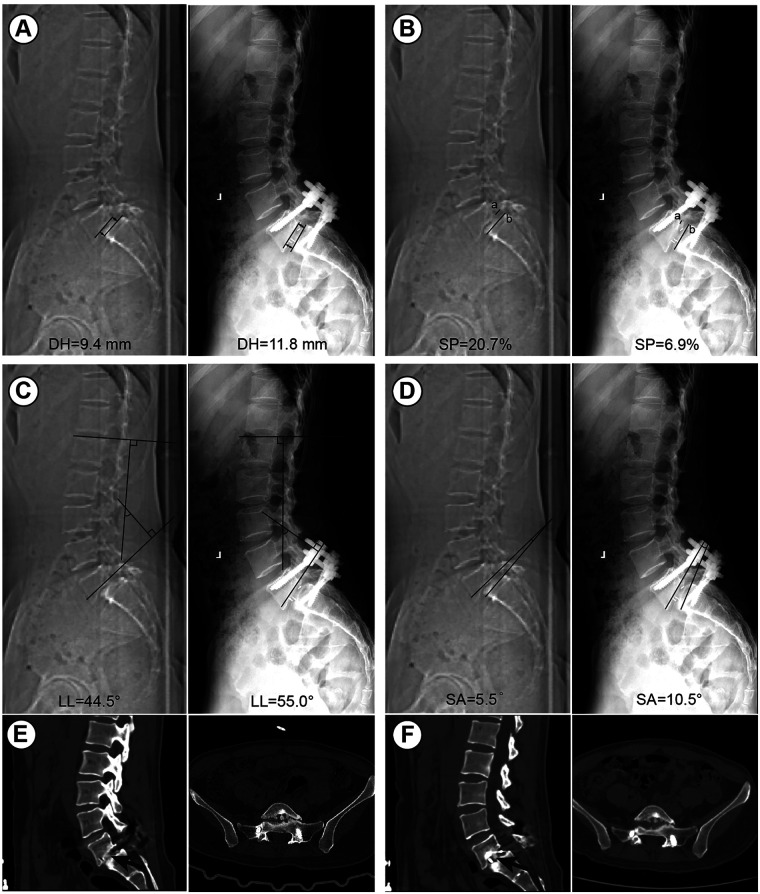
Radiological assessment of lumbar parameters and interbody fusion outcomes in a representative patient. **(A)** Measurement of DH on lateral radiographs. DH increased from 9.4 mm preoperatively to 11.8 mm; **(B)** measurement of SP, which decreased from 20.7% preoperatively to 6.9% postoperatively; **(C)** measurement of LL using the Cobb method. LL improved from 44.5° preoperatively to 55.0°; **(D)** measurement of SA, which changed from 5.5° preoperatively to 10.5°; **(E,F)** postoperative CT images obtained at 3 months **(E)** and 12 months **(F)** demonstrated successful interbody fusion at the operated segment. According to the Bridwell interbody fusion grading system, the fusion was classified as Grade II.

**Figure 6:**
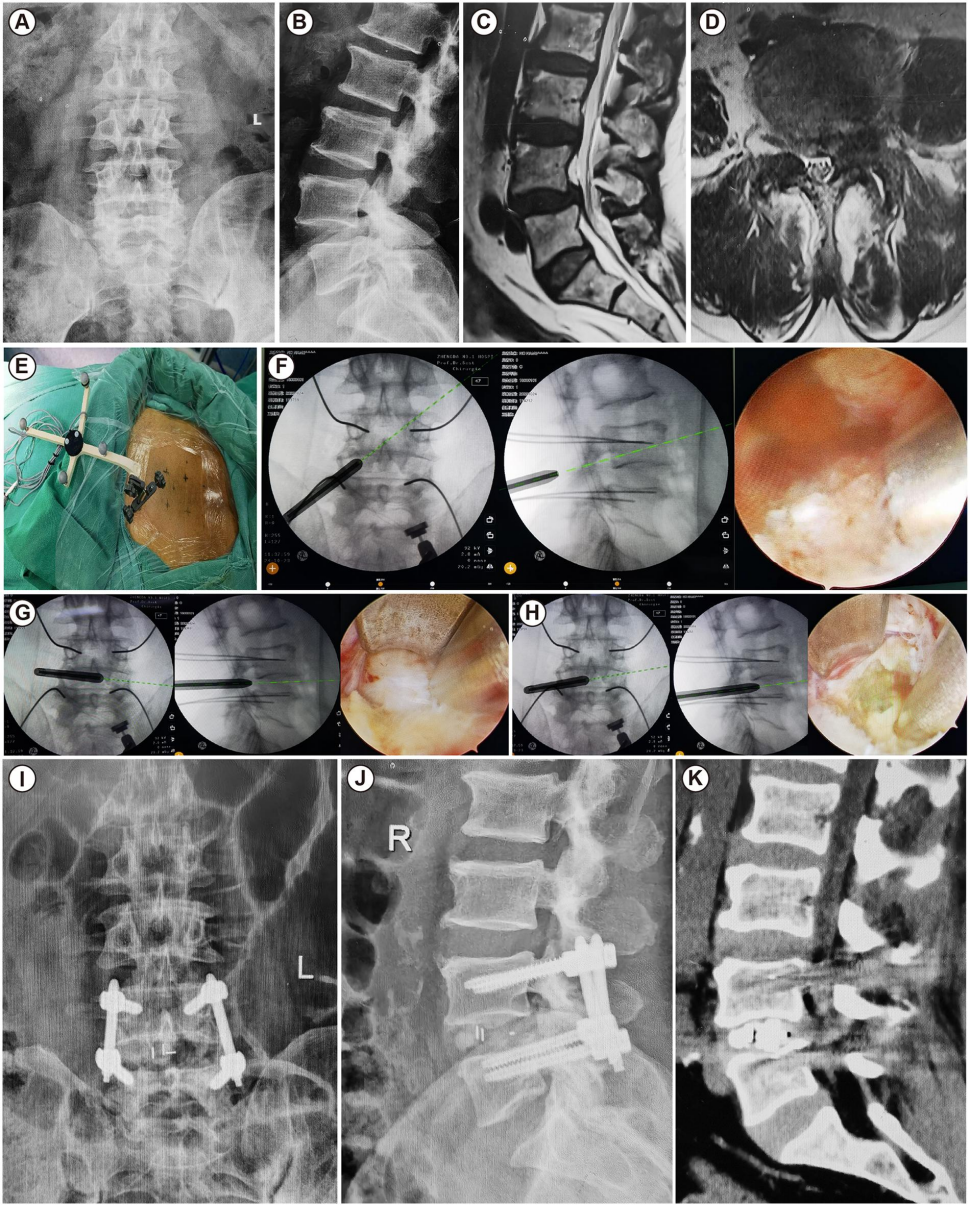
68-year-old male, with 1 year of lower back pain and left lower limb numbness, diagnosed with L4/5 lumbar spondylolisthesis, underwent L4/5 interbody fusion surgery. **(A,B)** Preoperative x-ray anteroposterior and lateral views of the patient; **(C,D)** preoperative lumbar MRI sagittal and axial images; **(E)** installation of the image-guided dynamic navigation reference frame; **(F)** dissect soft tissue to create the surgical space, identify the inferior articular process under the endoscope, and confirm with navigation and C-arm; **(G)** address the L4/5 intervertebral disc; **(H)** implant the interbody fusion cage; **(I–K)** postoperative review of lumbar x-ray anteroposterior and lateral views and lumbar CT shows good positioning of the implants.

In terms of postoperative Bridwell interbody fusion rate grading (Table 1), the navigation group had 16 cases at grade Ⅰ, 12 cases at grade Ⅱ, and 4 cases at grade Ⅲ. The non-navigation group had 18 cases at grade Ⅰ, 15 cases at grade Ⅱ, 5 cases at grade Ⅲ, and 1 case at grade Ⅳ, resulting in fusion rates of 87.5% and 84.6%, respectively, with no statistically significant difference (*P* > 0.05). As shown in Table 5, there were no statistically significant differences in DH, SP, LL, and SA preoperatively (*P* > 0.05). At the final follow-up, both groups showed an increase in DH and LL compared to preoperative values (*P* < 0.05), while SP and SA decreased (*P* < 0.05); however, there were no statistically significant differences between the two groups (*P* > 0.05) (Figure 5). A typical case is shown in Figure 6.

**Table 5:** Comparison of imaging parameters between two groups of patients.

Variable	Group	Preoperative	Last follow-up	Difference	Statistic
DH (mm)	Navigation (*n* = 32)	8.8 ± 1.5	11.4 ± 1.5	2.5 ± 0.6	*t* = −23.817; *P* < 0.001
	Non-navigation (*n* = 39)	8.7 ± 1.6	11.3 ± 1.7	2.6 ± 0.7	*t* = −53.944; *P* < 0.001
	Statistic	*t* = 1.024 *P* = 0.310	/	*t* = −0.313 *P* = 0.756	
SP (%)	Navigation (*n* = 32)	18.2 ± 2.9	5.4 ± 1.6	12.8 ± 1.8	*t* = 88.936; *P* < 0.001
	Non-navigation (*n* = 39)	18.1 ± 2.7	5.3 ± 1.5	12.8 ± 1.7	*t* = 107.990; *P* < 0.001
	Statistic	*t* = 0.163 *P* = 0.871	/	*t* = 0.185 *P* = 0.854	
LL (°)	Navigation (*n* = 32)	46.0 ± 5.6	52.2 ± 2.8	6.2 ± 1.1	*t* = −31.887; *P* < 0.001
	Non-navigation (*n* = 39)	45.1 ± 2.5	52.5 ± 2.5	6.4 ± 1.7	*t* = −34.188; *P* < 0.001
	Statistic	*t* = −1.139 *P* = 0.890	/	*t* = −1.802 *P* = 0.426	
SA (°)	Navigation (*n* = 32)	5.1 ± 1.3	10.4 ± 1.6	5.3 ± 1.6	*t* = −48.636; *P* < 0.001
	Non-navigation (*n* = 39)	5.2 ± 1.5	10.0 ± 1.5	5.2 ± 1.6	*t* = −48.665; *P* < 0.001
	Statistic	*t* = −0.273 *P* = 0.785	/	*t* = 0.402 *P* = 0.689	

## 4 Discussion

In recent years, the field of digital orthopedics has advanced rapidly, driving significant transformation in orthopedic care. As a key component of digital orthopedics, intraoperative navigation technology has enhanced the precision of surgeries. In spinal, joint, and bone tumor procedures, navigation systems provide real-time guidance for accurate positioning and implant placement. By integrating preoperative imaging with real-time data, surgeons can navigate complex anatomical structures more effectively, thereby reducing the risk of complications (26–28).

We utilized a light-magnetic integrated intraoperative navigation system-assisted ULIF surgery to treat patients with LS. This system is a simple and efficient C-arm-based two-dimensional computer navigation system that is easy to operate, has a low learning curve, and does not require high-end equipment. This technology reconstructs the surgical site using intraoperative C-arm imaging and automatically matches the images to the navigation system, eliminating the need for manual point and surface registration. Although the images may be less clear than CT images, this approach mitigates the impact of positional changes and other human factors on the accuracy of three-dimensional navigation during surgery, and it addresses the discrepancies that can arise from manual point selection and matching failures (29). The use of computer navigation has overcome the limitations of traditional x-ray fluoroscopy. By performing preoperative scans and reconstructing images, it can provide real-time two-dimensional images during surgery. Through virtual imaging overlay and localization techniques, the system allows for real-time monitoring of the position of surgical instruments and their distance to surrounding tissues, significantly enhancing surgical precision and safety. Additionally, it reduces the need for repeated fluoroscopy on the patient, leading to increased application in the field of orthopedic surgery. With the navigation system, surgeons can determine the optimal entry points and trajectories for screws, as well as select the most suitable screws, greatly reducing the risk of damaging blood vessels and nerves (30). Additionally, this navigation system is primarily based on C-arm technology, which means it has relatively low equipment requirements, allowing most hospitals with existing facilities to meet the necessary conditions for its use. Currently, surgical navigation systems can be categorized into three main types based on their positioning and tracking technologies (15, 31, 32). This navigation system combines the precision and stability of traditional optical navigation systems with the high accuracy of magnetic navigation features, integrating optical and electromagnetic navigation technologies to complement each other's strengths and allow for seamless switching between light and magnetic navigation during surgery. However, there are some limitations, such as the occurrence of drift during operation, which necessitates improvements in the instruments and surgical techniques. Additionally, specific surgical tools compatible with the navigation system need to be prepared in advance, and there is a learning curve associated with becoming proficient in using the navigation system. If these limitations can be addressed, the optical-magnetic integrated navigation system may fully demonstrate the advantages of computer-assisted navigation technology in the field of spinal surgery (16, 33). In addition, this system does not rely on intraoperative CT, O-arm, or robotic platforms and is fully compatible with standard C-arm equipment already available in most primary and secondary hospitals. Its workflow is straightforward, the learning curve is relatively low, and no additional high-end infrastructure is required. From a clinical application perspective, the navigation system provides clear real-time guidance for pedicle screw entry points and trajectories, which is particularly valuable for surgeons in primary hospitals with limited experience in endoscopic fusion procedures. By reducing reliance on repeated fluoroscopic examinations and subjective anatomical judgment, the system helps improve procedural safety, consistency, and operational efficiency.

In this study, we compared the clinical data of two groups of patients with LS undergoing ULIF surgery, with and without navigation, to explore their clinical treatment outcomes. We selected two groups with comparable patient profiles for analysis. The results showed that the duration of surgery in the navigation group was significantly shorter than in the non-navigation group. This suggests that, compared to traditional x-ray-assisted ULIF surgery, which has a steep learning curve, the use of an intraoperative navigation system can substantially reduce the time surgeons spend identifying the complex anatomical structures of the spine. For patients, this translates into shorter anesthesia times, thereby reducing surgical risks. Additionally, the number of C-arm fluoroscopy instances required during surgery to verify the proper positioning of the pedicle screws was significantly lower in the navigation group. This reduction in fluoroscopy led to a decrease in radiation exposure during the procedure. For patients, the radiation dose from a single surgery is well below the annual threshold that poses a risk for radiation-induced diseases, making the risk relatively low. However, for the surgical team, prolonged exposure to radiation, even with lead aprons for protection, may increase the risk of cancers and other diseases, highlighting the importance of minimizing radiation exposure (34). Additionally, intraoperative blood loss and postoperative drainage volume were lower in the navigation group compared to the non-navigation group. This may be attributed to the use of the navigation system, which reduced the time surgeons spent identifying optimal entry points and trajectories for screws, thereby minimizing the risk of vascular injury. With regard to intraoperative blood loss assessment, although conventional methods remain applicable in open surgical procedures, they tend to overestimate actual blood loss in endoscopic surgeries involving continuous saline irrigation. In contrast, the calculation-based method using the difference between irrigation volume and collected fluid provides a more practical approximation under these conditions. Nevertheless, this blood loss estimation technique has inherent limitations and has not yet been widely validated in lumbar fusion surgery. Therefore, in the present study, blood loss was interpreted primarily as a relative rather than an absolute parameter, and related comparisons were restricted to between-group analyses performed under identical operative conditions. These findings further demonstrate the precision and safety of ULIF surgery when guided by navigation (16). However, this study found no significant difference in the complication rates. This may be partly due to the insufficient sample size and the fact that we did not categorize the types of postoperative complications in detail, only recording major complications (35). Additionally, there were no statistically significant differences in postoperative hospital stay, Bridwell interbody fusion rates, postoperative leg pain VAS scores, or ODI scores between the two groups.

However, we observed that during the early postoperative follow-up period, patients in the navigation group exhibited significantly lower VAS scores for low back pain compared with those in the non-navigation group, and this difference reached statistical significance. We speculate that this difference may be attributed to several factors. First, navigation-assisted surgery enables more accurate pedicle screw placement and more optimal cage positioning, which may contribute to improved segmental alignment and load distribution at the surgical level. Even when screw trajectories or cage positions are considered clinically acceptable, minor deviations may result in subtle instability or asymmetric stress distribution during early postoperative mobilization, potentially exacerbating postoperative pain. Second, navigation technology reduces reliance on repeated fluoroscopic confirmation and intraoperative adjustments, thereby streamlining the surgical workflow. The decreased need for repeated instrumentation and repositioning may help minimize paraspinal muscle retraction, facet joint disturbance, and iatrogenic soft tissue injury, all of which are recognized contributors to early postoperative low back pain. Third, the increased surgical confidence afforded by navigation assistance may allow for more controlled decompression and instrumentation, potentially reducing inadvertent endplate violation or excessive bony manipulation, factors that have been associated with postoperative discomfort and pain. We also acknowledge that early postoperative pain is multifactorial in nature and may be influenced by potential confounding factors such as individual pain perception, the extent of muscle injury, and subtle differences in intraoperative handling, which are difficult to quantify in a retrospective study. This represents one of the limitations of the present study. Additionally, this study is a retrospective analysis with a limited number of cases and a short follow-up period. Future large-sample randomized controlled trials with extended follow-up are needed for further evaluation of the treatment effectiveness of this technique.

## 5 Conclusion

In summary, ULIF surgery for LS, performed with this simple and efficient navigation system, can achieve favorable early outcomes. The duration of surgery, number of fluoroscopy instances, postoperative drainage volume, and intraoperative blood loss were all lower in the navigation group compared to traditional x-ray-assisted ULIF surgery, and patients showed more significant improvements in early postoperative VAS scores for low back pain. However, in the long term, there were no differences in interbody fusion rates, LL, SA, SP, and DH between patients undergoing ULIF surgery with navigation and those undergoing traditional x-ray guidance.

## Data Availability

The raw data supporting the conclusions of this article will be made available by the authors, without undue reservation.
